# Supplementary material for: Introduction of a More Glutaredoxin-like Active Site to PDI Results in Competition between Protein Substrate and Glutathione Binding
Source: Antioxidants (Basel). 2022 Sep 28;11(10):1920. doi: 10.3390/antiox11101920 (PMC9598436; doi:10.3390/antiox11101920)
Supplement: Supplementary file 1 [file antioxidants-11-01920-s001.zip › antioxidants-1938709-supplementary.pdf]

antioxidants

MDPI

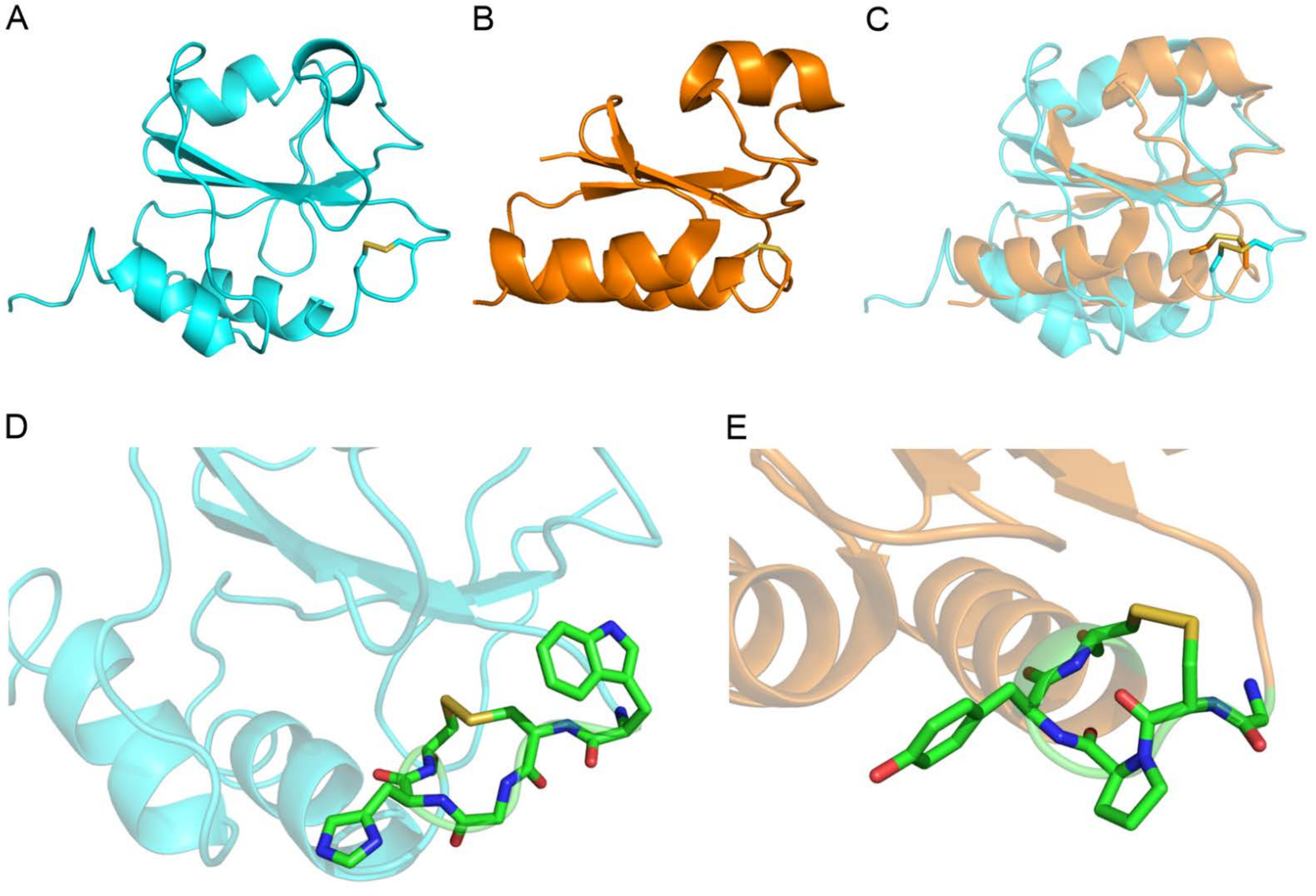

Figure S1. NMR Structures of human PDI and *E.coli* Grx1. NMR structures of (**A**) human PDI **a** domain (1MEK) and (**B**) *E.coli* Grx1 (1EGO). Panel (**C**) shows superimposition of the structures demonstrating the similarity of the thioredoxin fold structure found in both. The active sites of (**D**) human PDI **a** domain (WCGHC) and (**E**) *E.coli* Grx1 (GCPYC) differ from each other.

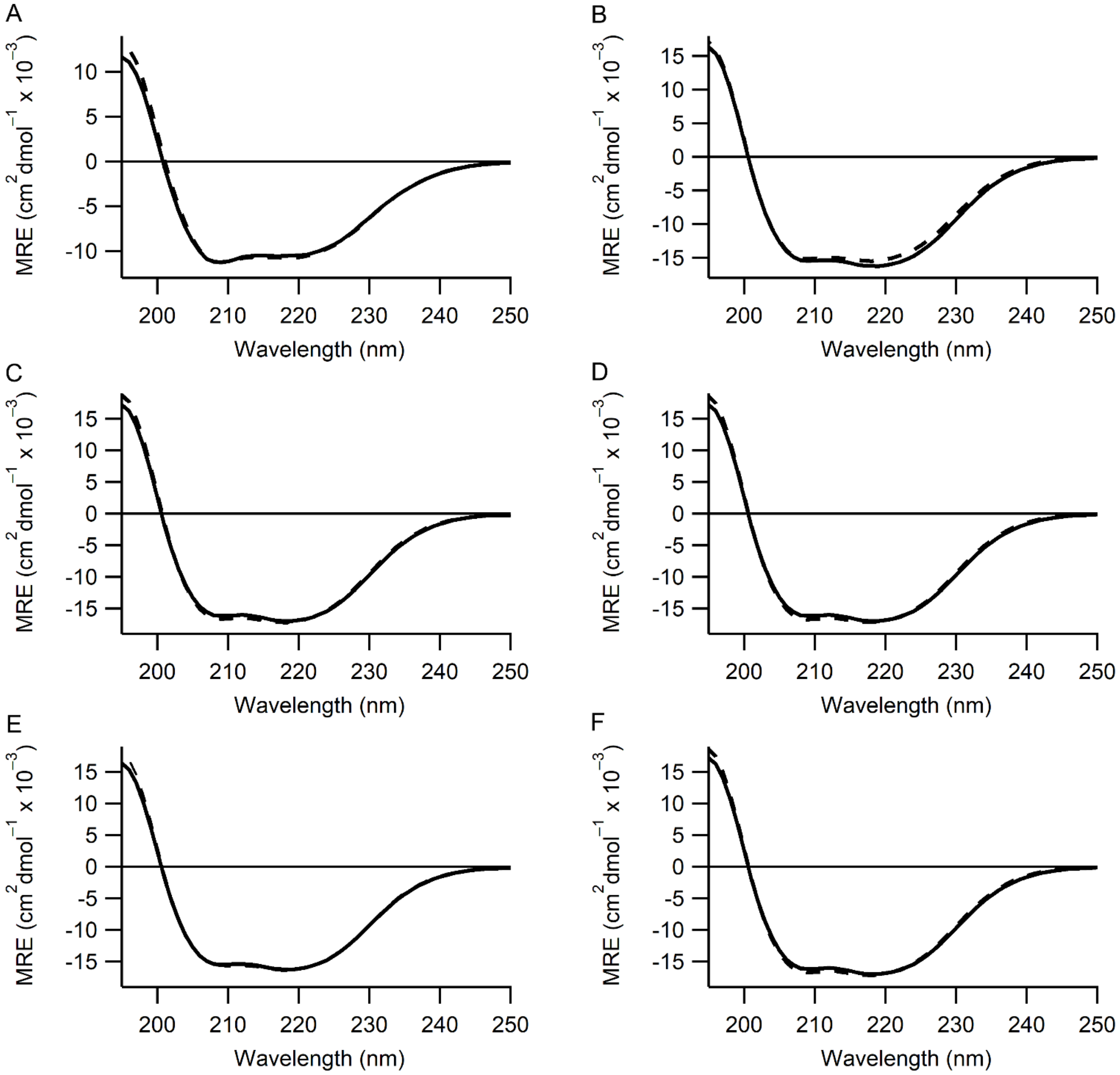

Figure S2. PDI variants have the same secondary structure as wild-type. Far UV CD spectra for (**A**) wild-type (solid line) and H55F H399F (dashed line) mature PDI, (**B**) wild-type (solid line) and H55F (dashed line) **a** domain of PDI, (**C**) wild-type (solid line) and G54N H55F (dashed line) **a** domain of PDI, (**D**) wild-type (solid line) and G54P H55F (dashed line) **a** domain of PDI, (**E**) wild-type (solid line) and G54Q H55F (dashed line) **a** domain of PDI and (**F**) wild-type (solid line) and G54S H55F (dashed line) **a** domain of PDI. Average traces of at least 3 separate CD spectroscopic scans are shown. MRE = mean residue ellipticity.

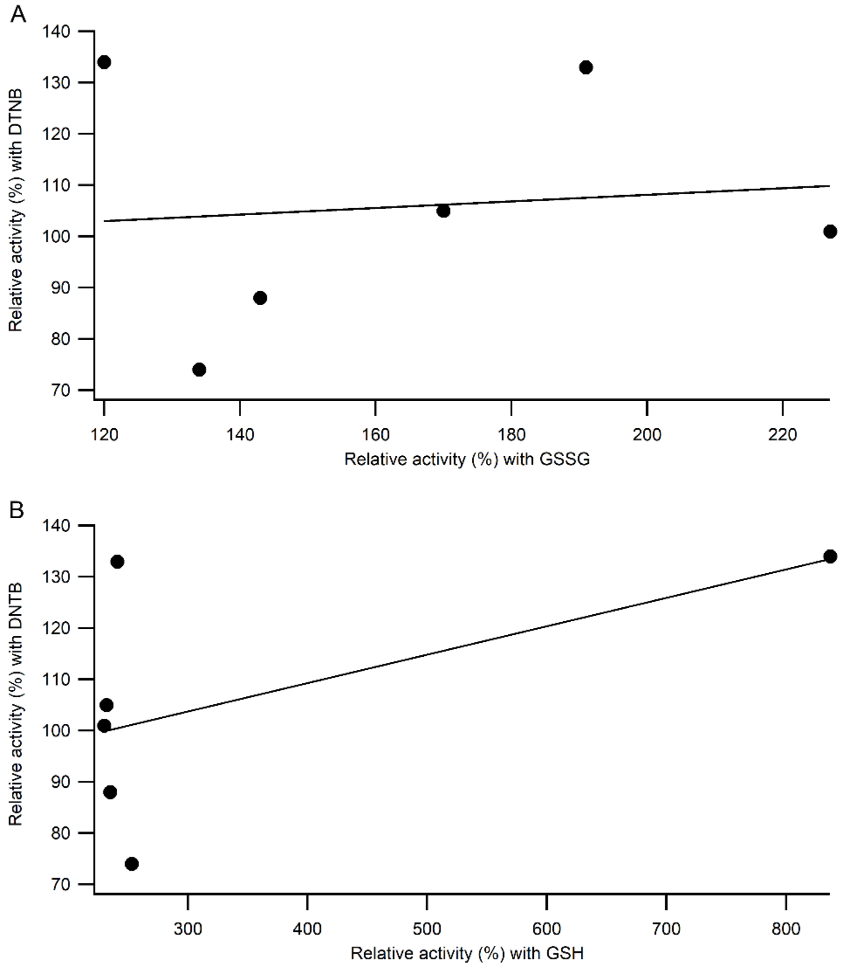

Figure S3. Correlation of relative activity of PDI **a** domain variants with DTNB and GSSG or GSH. No correlation was observed between relative activity of the PDI a domain active site variants with DTNB and GSSG (panel **A**, $R^2 = 0.0114$) or GSH (Panel **B**, $R^2 = 0.318$).
